# Comment on ‘Systematic Druggable Genome‐Wide Mendelian Randomization Identifies Therapeutic Targets for Sarcopenia’ by Yin Et Al.

**DOI:** 10.1002/jcsm.13589

**Published:** 2024-11-19

**Authors:** Tianrui Liu, Feixiang Yang, Kun Wang, Peng Guo, Jialin Meng

**Affiliations:** ^1^ Department of Urology, The First Affiliated Hospital of Anhui Medical University; Anhui Province Key Laboratory of Urological and Andrological Diseases Research and Medical Transformation Anhui Medical University Hefei China; ^2^ Second School of Clinical Medicine Anhui Medical University Hefei China; ^3^ Department of Urology The Affiliated Jiangyin Hospital of Nantong University Wuxi China

Dear Editor,

We recently read with great interest the paper by Yin and colleagues [1] that pharmacologically available genomic data, cis‐eQTL/cis‐pQTL from human blood and skeletal muscle tissue, and GWAS pooled data on sarcopenia related traits were used to analyse the potential causal relationship between drug target genes and sarcopenia. The study employed colocalization and Mendelian randomization (MR) analyses to identify 17 potential therapeutic targets for sarcopenia. However, in the articles by Yin et al. [2], they put forward their own views on this method of analysis. Colocalization analysis after MR analysis may introduce uncorrelated pleiotropic effects due to violation of the exclusion–restriction assumption, which cannot strengthen the MR results.

The thriving development of genome‐wide association studies (GWAS) over the past decades has laid the foundation for the explosive growth of MR studies in recent years. Recently, an increasing number of MR studies have begun to explore the efficacy and safety of novel drugs and to seek new applications for existing drugs [3]. Colocalization methods were invented to discover how disease‐associated genetic variants revealed by GWAS affect downstream pathways and whether genetic variants may share one or more causal variants with potential biological mediators; they are commonly used as sensitivity analyses after MR analysis to strengthen the reliability of the results [4]. Identifying and classifying pleiotropy is a crucial step in MR research, which can be divided into vertical pleiotropy and horizontal pleiotropy, with horizontal pleiotropy further divided into uncorrelated horizontal pleiotropy and correlated horizontal pleiotropy. In MR studies, when the exposure of genetic instrumental variable (IV) affects the outcome through its impact on downstream traits, it is vertical pleiotropy, representing the essence of the MR methodology [5]. Moreover, when exposure IV not only influences the outcome through exposure but also affects the outcome through common confounding factors between exposure and outcome, it is referred to as correlated horizontal pleiotropy. However, uncorrelated horizontal pleiotropy occurs when other genetic variations (such as single nucleotide polymorphisms, SNPs), due to linkage disequilibrium (LD), collectively impact the outcome alongside the exposure to genetic IV [6].

Although uncorrelated horizontal pleiotropy can be addressed by methods such as MR‐Egger or MR‐PRESSO, it may still introduce potential biases to MR results. In cis‐MR, colocalization between expression quantitative trait locus (eQTL) and GWAS data suggests the sharing of a genetic locus between upstream genes and downstream traits, indicating vertical pleiotropy [4]. This strengthens the causality in MR and contradicts Yin's claim of violating the third assumption. However, in polygenic MR analysis [7], the colocalization of GWAS with GWAS data indicates the sharing of a genetic locus between two phenotypes. This implies the existence of uncorrelated horizontal pleiotropy, violating the exclusion–restriction assumption, thus undermining support for MR results. After detecting horizontal pleiotropy through the MR‐Egger intercept test, the MR pleiotropy residual sum and outlier (MR‐PRESSO) test, or other methods, the CAUSE method can be used to comprehensively consider horizontal pleiotropy [6]. It can determine whether the pleiotropy between exposure and outcome is uncorrelated horizontal pleiotropy or correlated horizontal pleiotropy, thus cautiously interpreting the MR results.

Employing the CAUSE methodology enables researchers to interpret MR outcomes with increased caution, identifying the specific type of pleiotropy influencing the linkage between exposure and outcome. This enhancement in precision and trustworthiness of MR investigations is particularly significant amidst intricate biological pathways and genetic mechanisms. Therefore, the CAUSE method provides a powerful tool for MR analysis, which allows for a comprehensive consideration of horizontal pleiotropy when detected, thus inferring causal relationships more accurately. MR and colocalization indeed occupy critical roles within the fields of genetic epidemiology and genomics research, and the result of MR is typically interpreted as the causal relationships between exposure and outcome; colocalization results are used to unveil genetic architectures and biological mechanisms [4]. Despite differences in concept and practice between MR and colocalization analysis, both approaches leverage genetic variations to investigate the relationships among traits, serving as critical tools in contemporary genetic epidemiology.

Lately, with the publication of high‐quality GWAS and eQTL data, the transcriptome‐wide association studies (TWAS) approach has been extensively utilized to uncover the connections between gene expression levels and complex traits. TWAS employs eQTL data to build predictive models of gene expression, which are then utilized to evaluate the relationship between gene expression levels and traits. Through this method, researchers can indirectly assess the impact of gene expression on traits without directly measuring gene expression levels. The majority of trait‐associated genes identified through TWAS are physically well separated from other candidate genes; thus, they are less influenced by LD than those identified by GWAS [8]. Cis‐MR and TWAS are both methods that utilize genetic variations to study the association between gene expression and traits. They share conceptual similarities, particularly in using genetic variations to infer potential causal relationships between gene expression and traits. The advancement of TWAS research provides new tools and algorithms for MR studies. When conducting MR research, we can use the latest TWAS algorithms such as MR‐JTI [9] and cTWAS [10] to support our MR results. These algorithms offer a robust approach to enhance our causal inference regarding the association between gene expression levels and traits. By integrating the use of these methods, researchers can validate their findings from various angles, thereby enhancing the credibility and precision of causal inference.

Therefore, based on the above discussion, we recommend that in conducting MR analysis, if conducting cis‐MR studies, leveraging colocalization findings can bolster conclusions, supplemented by TWAS analysis to enhance the reliability of MR results. Whereas in polygenic MR analysis, we should cautiously interpret colocalization results, employ multiple analytical methods for cross‐validation, such as CAUSE methods, and conduct analysis as per the requirements of STROBE‐MR guidelines.

## Conflicts of Interest

The authors declare no conflicts of interest.

## Data Availability

The authors have nothing to report.
